# Supplementary material for: SNRAware: Improved Deep Learning MRI Denoising with SNR Unit Training and G-factor Map Augmentation
Source: ArXiv. 2025 Mar 23:arXiv:2503.18162v1. Preprint. [Version 1] (PMC12306816)
Supplement: Supplement 1 [file NIHPP2503.18162v1-supplement-1.pdf]

Supplemental Appendices

Appendix E1. Information for deep learning models

As shown in Figure 2, the model consists of three components: pre-convolution layer, backbone and post-convolution layer. The input tensors are in the shape of [B, C, T/S/D/Z, H, W]. C is 3 for complex inputs (real, imagery and g-factor). Noise in the input images are scaled to 1.0×g-factor, as this setup is consistent with reconstruction outputs.

The pre-convolution layer is a shallow feature extractor (36). It is kept being minimal as a 2D convolution to uplift input channel C to 64, encouraging backbone to take on most heavy lifting and helping generalization. The post-convolution is another CONV layer, converting $C_{backbone}$ after the backbone to required output channels (2 for complex training and 1 for magnitude training).

Two well-known backbone architectures, HRnet and Unet, are implemented and tested in this study. Both architectures utilize the multi-resolution pyramid to balance model size, expressive power and computing cost. The building components include multiple Blocks, downsample and upsample layers, channel-wise concatenation, and skip connection. The HRnet maintains a longer pipeline on the original tensor size and Unet is smaller in size and less computing expensive.

The input tensors are processed through every block, gaining more channels and reducing spatial resolution, which is explained by the backbone plots annotated with tensor sizes.

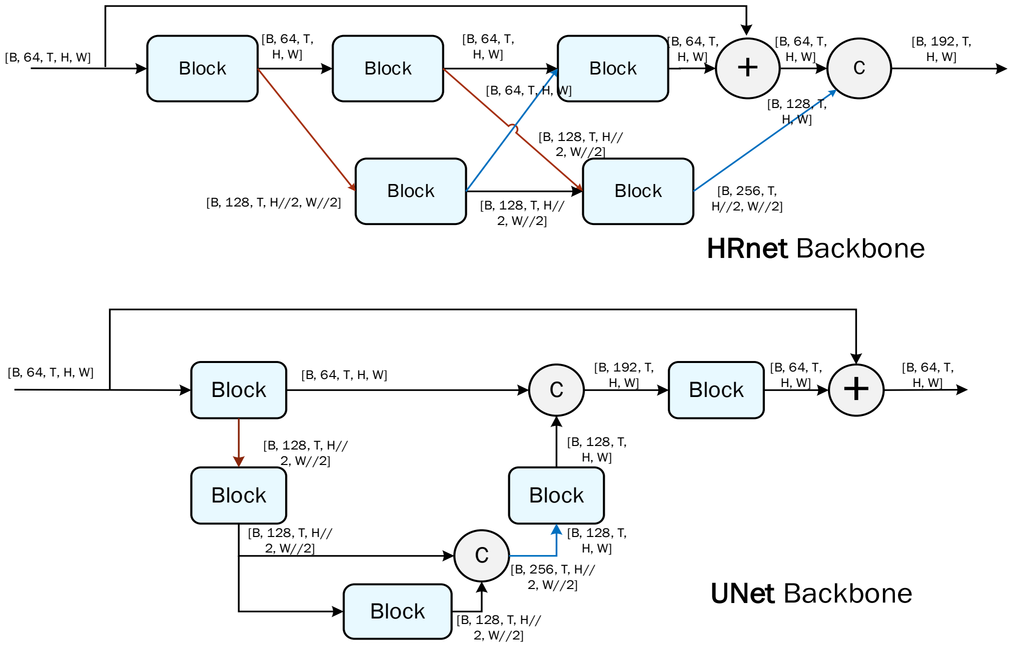

Figure 1. Annotated backbone architectures.

Downsampling was implemented with patch merging (24) followed by a convolution to format outputs to have the required number of channels. The upsampling was implemented with a linear interpolation followed by a CONV layer.

Backbones consist of several blocks. A block is a container of N cells. Every cell has a classical setup of two skip connections, layer norms (37) and attention or convolution layers.

By switching the attention methods (e.g. Swin3D, ViT3D or CNNT etc.), we can instantiate different models for experiments. A pure convolution model was implemented by replacing attention with convolution layers.

Every block in all models, except CNNT-large, has 3 cells. For CNNT-large, a block holds 6 blocks. By inserting more cells or more blocks, the model can be scaled up or down.

As used in other denoising training schemes, models were trained on image patches to encourage models to focus on noise distribution instead of image content. The patch size was [T/S/D/Z=16, H=64, W=64]. The window size in Swin3D and ViT3D was [16, 8 ,8], where every [2, 2, 2] neighborhood was processed as a token. The CNNT transformer method computed attention between all [H, W] frames without explicit neighborhood tokenization. All convolutions had the kernel size 3 and padding 1. We note that unlike the original Swin and ViT papers, we re-patch and un-patch the tensors before and after every operation, resulting in imaging tensors that can be processed by the normalization and convolutional mixer layers in every cell.

Supplemental Data

Movie 1: The movies correspond to the example in Figure 1b. The ground-truth clean image is the single one on the left. The first row are the noisy samples. The second row are the SNR images.

Movie 2: Corresponding movies to Figure 4 are given here.

Movie 3: More R=5 real-time cine examples are given here. In all cases, proposed training noticeably improves performance. The leftover noise amplification is very visible without the g-factor map.

Movie 4: Movies of perfusion denoising corresponding to Figure 5 are presented. Model generalized well to dynamic contrast and low base SNR.

Movie 5: Movie corresponds to Figure 6a for the T1 MPRAGE neuro test.

Movie 6: Movie corresponds to Figure 6b for the T2 TSE spine test.
